# Synergistic Electric and Thermal Effects of Electrochromic Devices

**DOI:** 10.3390/mi13122187

**Published:** 2022-12-10

**Authors:** Meng Yuan, Hanlin Yin, Yitong Liu, Xiaohua Wang, Long Yuan, Yu Duan

**Affiliations:** 1 College of Science, Changchun University of Science and Technology, Changchun 130012, China; 2 Key Laboratory of Functional Materials Physics and Chemistry of the Ministry of Education, Jilin Normal University, Changchun 130012, China; 3 State Key Laboratory of Integrated Optoelectronics, College of Electronic Science & Engineering, Jilin University, Changchun 130012, China

**Keywords:** electrochromic, thermal effect, synergistic, temperature

## Abstract

Electrochromic devices are the preferred devices for smart windows because they work independently of uncontrollable environmental factors and rely more on the user’s personal feelings to adjust actively. However, in practical applications, the ambient temperature still has an impact on device performance, such as durability, reversibility and switching performance, etc. These technical issues have significantly slowed down the commercialization of electrochromic devices (ECDs). It is necessary to investigate the main reasons for the influence of temperature on the device and make reasonable optimization to enhance the effectiveness of the device and extend its lifetime. In recent years, with the joint efforts of various outstanding research teams, the performance of electrochromic devices has been rapidly improved, with a longer lifetime, richer colors, and better color contrast. This review highlights the important research on temperature–dependent electrochromic properties in recent years. Also, the reported structures, mechanisms, characteristics, and methods for improving electrochromic properties are discussed in detail. In addition, the challenges and corresponding strategies in this field are presented in this paper. This paper will inspire more researchers to enrich the temperature–dependent properties of ECDs and their related fields with innovative means and methods to overcome the technical obstacles faced.

## 1. Introduction

**Figure 1:**
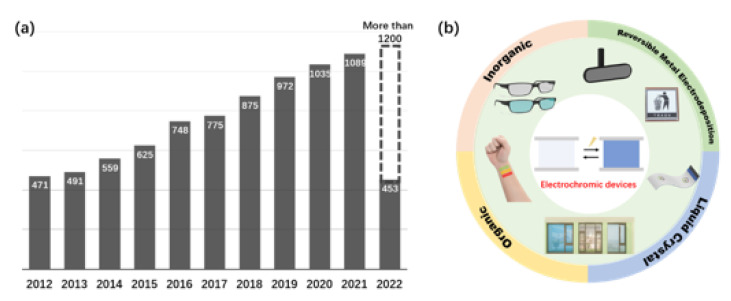
(**a**) quantity of published articles about ECDs in the last ten years retrieved on the web of science. (**b**) applications of ECDs with different systems.

Solar energy is the most important source for mankind. Therefore, improving the efficiency of solar energy utilization is one of the most important concerns of modern researchers [1]. Smart windows reversibly switch between transparent and blocked states to dynamically control the transmission of solar radiation into buildings [2]. Reduces glare and provides an unobstructed view and natural daylighting while reducing energy consumption for heating and cooling. Smart windows can be divided into three main categories according to their operating principles: electrochromic windows change transmittance under applied voltages, photochromic windows respond to environmental stimuli by altering their transmittance with the change of light intensity, and thermochromic windows by temperature. Compared to photochromic and thermochromic smart windows, the biggest advantage of electrochromic smart windows is their ability to provide dynamic modulation across the spectrum and active control based on individual user preferences, while the other two can only work passively depending on the light intensity and temperature in the environment. In Figure 1a, the number of articles about electrochromic devices (ECDs) searched on the Web of Science in the past decade. We can observe that the research on electrochromic devices has been on the increase every year, which indicates that the research on ECDs has received wide attention from researchers.

As shown in Figure 1b, with the advancement of research, ECDs have been applied in more fields in recent years (such as anti–glare rear–view mirrors for automobiles, paper–like electronic devices for eye–friendly and low energy consumption displays, etc.) [3,4,5,6], demonstrating the greater potential application. Conventional ECDs usually have a multilayer structure, including an electrochromic functional layer, an ion storage layer for charge balancing, an ion transport layer, and conductive electrodes on both sides [7,8,9]. As we all know, the operation of the ECD depends on the corresponding redox reaction of the functional layer under the action of an external voltage. Moreover, the temperature will play a very important influence on this electrochemical reaction. In practical applications, the temperature also affects many properties of the device [10].

The effect of temperature on the device consists of three main components. They have been categorized here as: (1) temperature effects on the morphology and crystal structure of electrochromic materials. It mainly occurs at the stage of preparation of the functional layer of the device and the effect of temperature on it when the device is in operation. (2) The effect of temperature on the energy required for electrochemical reactions. This part is mainly the effect on the activation energy required for the reaction and/or the ratio of activation factors. (3) The ability of the electrochromic device to regulate the temperature of the environment. The ability of different device types to modulate the infrared band and their ability to modulate the indoor dimension is mainly discussed.

Herein, we first introduced the operating principles and equipment structures of several typical ECDs and their characteristics in Section 2. The focus is on the effect of temperature on the electrochromic performance of the device in Section 3. At the same time, it summarizes and concludes the work of the other three parties in recent years in Section 4, Section 5 and Section 6. Although these studies have been neglected in previous reviews, we believe they are equally important, and their valuable results should be communicated to those involved in the development of ECD and other devices. Finally, we offer our perspective on the challenges and opportunities for future research. We hope this progress report will help readers to get a comprehensive understanding of the latest research progress in ECDs. Moreover, how can we consider minimizing the negative impact of temperature, an uncontrollable condition, on their performance when facing applications? The performance and lifetime of the devices can be improved by proper device structure design.

## 2. Typical Electrochromic Devices

The transmittance, reflectance, absorptivity, and emissivity of ECDs can be dynamic accommodation [11]. Many types of systems are known to exhibit differing degrees of electrochromic effects. ECDs, depending on the reaction type of electrochromism possess, can be grouped into four categories: inorganic systems, organic systems, liquid crystal based Electrochromic systems (LC), and reversible metal electrodeposition–based electrochromic systems (RME). The advantages of inorganic systems are good stability and reliability, but the problems associated with monotonous colors, slow response times and high fabrication costs [12,13]. In contrast, the properties of organic systems are opposite. They have high processability, high color versatility, and rapid response times but are inferior in terms of thermal resistance, chemical stability, and device durability, generally [14,15]. For the liquid crystal–based electrochromic systems, bulk electrochromism can occur in LCs without any other electrolyte, and they have rapid response times. However, its operating voltage is large, and the temperature stability is poor. It is different from other systems, and the RMEs are mainly used in variable reflectance mirrors [16,17] and light shutters [12,18] due to their super reflectivity adjustment. This broad variability in the characteristics and composition of ECDs provides a solid basis for the attempted fusing of electrochromic technology with other advanced technologies.

### 2.1. Inorganic Systems

Inorganic electrochromic materials mainly include transition metal oxides and metal hexacyanometallate–based systems. It has been widely studied for its excellent environmental stability and ease of fabrication in a large area.

**Figure 2:**
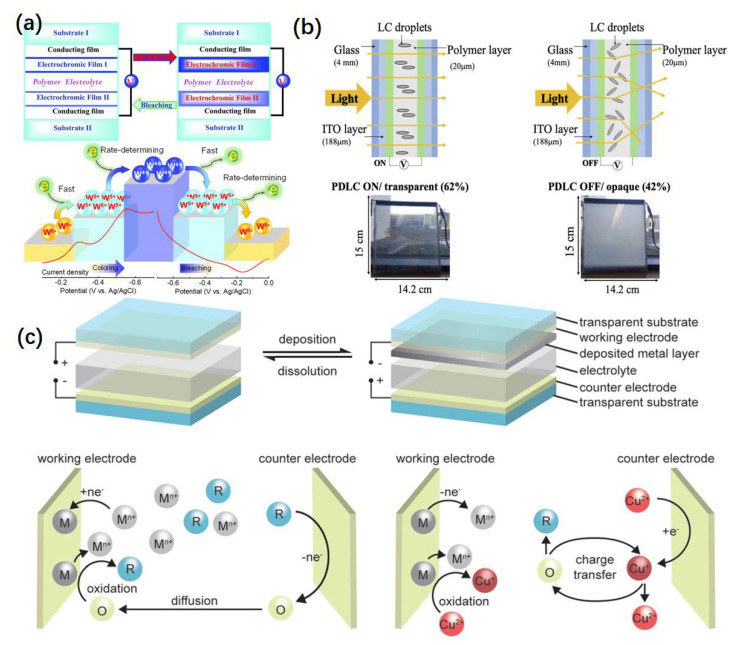
(**a**) Common structure and valence change of the central atom of conventional ECDs. Reprinted with permission from Ref. [19]. Copyright 2012, Wiley. Copyright and Licensing” are available via the following link: https://www.mdpi.com/ethics#10.Reprinted with permission from Ref. [65]. Copyright 2021, American Chemical Society. Copyright and Licensing” are available via the following link: https://www.mdpi.com/ethics#10. (**b**) The operating principle of PDLC and the sequential effect of changes. Reprinted with permission from Ref. [66]. Copyright 2020, Elsevier. Copyright and Licensing” are available via the following link: https://www.mdpi.com/ethics#10. (**c**) Component structure and operating principal diagram of an RME. Reprinted with permission from Ref. [67]. Copyright 2021, Wiley. Copyright and Licensing” are available via the following link: https://www.mdpi.com/ethics#10.

Although there are some studies on amorphous transition metal oxides, most of the studies are focused on crystalline transition metal oxides and the lattice structures, and most of them take the form of an octahedral geometry. Figure 2a shows the common structure and valence change of the central atom of conventional ECDs. The color change of these materials is mainly due to the charge transfer between the optical intervalence between the metal centers, and this process is accompanied by the insertion of anions or cations in the electrolyte into the lattice gap, respectively. Electrochromic that are colored in their reduced states are referred to as cathodically coloring. On the other hand, electrochromic that are colored in their oxidized states are anodically colored. A list of some of the more common transition metals, types of coloring, and their respective electrochromic color changes are given in Table 1.

The other important class of inorganic electrochromic materials is based on metal hexacyanometallates and their derivatives [46,47]. They are a group of mixed–valence compounds with the general formula M^1^_x_[M^2^CN_6_]_z_, where M^1^ and M^2^ refer to transition metal ions existing in different oxidation states. Moreover, the most established material is Prussian blue (iron hexacyanoferrate, Fe_4_[Fe(CN)_6_]_3_). They consist of electronically active metal sublattices and an open 3D framework structure built by a cyanide–bridged network of octahedral units [46]. The intense blue coloration of these systems is due to intervalence electron transfers between the mixed oxidation states of the iron atoms [48]. Prussian blue can exhibit both cathodic and anodic coloring. It appears green when oxidized and white when reduced. Its unique structural characteristics make its color change response fast, but the color change single disadvantage also still exists in these materials.

### 2.2. Organic Systems

The organic electrochromic materials mainly include viologens, metal–ligand complexes, and conjugated polymers.

Viologens are a class of 4,4–bipyrudine–containing compounds that can adopt three different redox states for radical dictation cation, a di–reduced neutral compound and accompanied by charge–balancing counter–anions correspondingly [49,50,51,52]. The most stable states and usually presents colorless unless it undergoes charge–transfer interactions with the ions. The advantage of viologens is that they can exhibit very high color contrast due to the special molar extinction coefficient in the radical and cationic forms, and they are readily available. However, viologens are usually operated in their solution, which may lead to leakage issues of the devices. Furthermore, a constant power supply is required to sustain such materials in any of their redox states.

**Figure 3:**
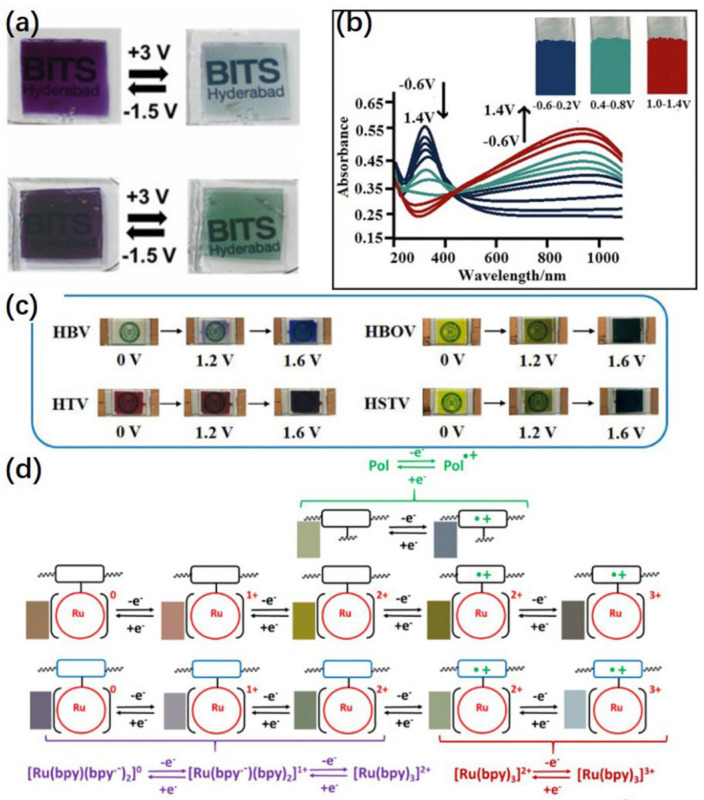
The performance of organic polymers of different materials in terms of color diversity. (**a**) The color change of polyFe and polyFe–QD–10–based ECDs upon applied voltage. Reprinted/adapted with permission from Ref. [77]. Copyright 2022, Elsevier”. More details on “Copyright and Licensing” are available via the following link: https://www.mdpi.com/ethics#10. (**b**) Spectroelectrochemical spectra of poly (3MT–co–EDOT) as applied potentials between 0.6–1.4 V in pH 7.0. Reprinted/adapted with permission from Ref. [78]. Copyright 2022, Elsevier”. More details on “Copyright and Licensing” are available via the following link: https://www.mdpi.com/ethics#10. (**c**) Color change of these four ECDs at different voltages. Reprinted/adapted with permission from Ref. [79]. 2022, Elsevier”. More details on “Copyright and Licensing” are available via the following link: https://www.mdpi.com/ethics#10. (**d**) Schematic representation of the redox process of the polymers. Reprinted/adapted with permission from Ref. [80]. 2022, Elsevier”. More details on “Copyright and Licensing” are available via the following link: https://www.mdpi.com/ethics#10.

Metal–ligand complexes are attractive electrochromic materials due to their intense, vivid colors with molar extinction coefficients. Figure 3 shows the performance of organic polymers of different materials in terms of color diversity. Due to the different types of metal elements, they can be divided into lanthanide–based ligand complexes and transition metal–ligand complexes. Lanthanide–based phthalocyanine complexes generally adopt a sandwich structure, while transition metal cations in transition metal phthalocyanines reside in the middle of the phthalocyanine ring to form a planar complex [53] as many of them have the potential to exhibit multichoice behaviors duo so they can undergo both oxidation and reduction processes [54,55,56,57]. The advantage of these materials is that the electrochromic response time is fast, and the electroactivity is more than 10^6^ cycles. However, they usually suffer from poor mechanical properties in their film states [58,59].

Conjugated polymers are also referred to as conducting polymers because of their ability to conduct electricity. They are macromolecular organic species whose backbones are built up by alternating double and single bonds. Conjugated polymers mainly include polythiophenes, polypyrroles, polyanilines, polyindole, polyfurans and polycarbazoles. Electrochemical doping can alter the structures of their π–conjugated systems, inducing changes in both their bandgaps and optical contrasts. They can be categorized into two groups, namely all–donor and donor–acceptor types. The namely all–donor consists of electron–donating monomers only in the chemical makeup, whereas the donor–acceptor is composed of both electron–donating and electron–accepting monomers that are usually linked in an alternating way [60]. Polymers have several advantages over other materials, such as high optical contrasts, rapid response times, and high coloration efficiencies than inorganic counterparts [61]. The films of polymers can very often be prepared from solution; the preparation and processing steps are relatively easy and cheap. Moreover, the ease of tailoring their color and properties through chemical structural modifications and the possibility of displaying multiple colors within a single material [62,63]. Polymers are flexible, and they can, therefore, be molded and bent into various shapes and be expected to use in future Integration with wearable technology, such as ranging from robotic skin, human–machine interfaces, energy devices for soft electronics and optoelectronic devices [5,64]. One major limitation is their shorter cycle lives due to the susceptibility of polymeric materials toward oxidative and UV degradation.

### 2.3. Liquid Crystal–Based Electrochromic Systems

Liquid crystal (LC), electrophoretic and electrochromic are three technologies of the passive display, which is low power [68]. The liquid crystal can change its color characteristics under the control of voltage; it is a separate electrochromic system in this paper. There are many kinds of liquid crystal, among which polymer dispersed liquid crystal (PDLC) is the most widely used as electrochromic [69]. Figure 2b shows the operating principle of PDLC and the sequential effect of changes. LC is dispersed in a transparent polymer matrix to form micron–sized or nano–sized liquid crystal droplets. Polymer provides a stable network structure for liquid crystal droplets, and its application properties strongly depend on the composition, morphology, size, interface properties, particle size distribution, and the matching of the properties of the two phases [70]. Due to the strong optical anisotropy and dielectric anisotropy of liquid crystal molecules, the material has remarkable electro–optical properties [71].

In the state of no electricity, the optical axis orientation of the LC droplet is random, and the refractive index of liquid crystal is anisotropic, so the light will scatter when it hits the PDLC film, and the PDLC film is opaque or translucent [72]. After electrifying, under the effect of enough electric field, the droplets of liquid crystal optical axis orientation in the direction of the electric field, as if the ordinary refractive index of liquid droplets and the refractive index of the polymer matrix, matching the PDLC film is transparent, after removal of an electric field, because the LC and interface reaction of polymer and the random orientation of LC droplets and restore to the original, the PDLC film also returns to an opaque state, thus completing an on–off conversion [73]. The advantage of LC–based electrochromic systems lies in their simple preparation, low cost and easy mass production [74]. However, as an electrochromic smart window, its low contrast and high driving voltage greatly limit its wide application, and the arrangement of uneven particles may lead to the formation of local hot spots that are the device [75,76].

### 2.4. Reversible Metal Electrodeposition–Based Electrochromic System

The RME system is a novel electrochromic application that utilizes the appearance and disappearance of a metal layer to achieve spectrum control. The metal cations deposit as metal particles or film on the electrode; this electrodeposition of metal induces the change from a transparent state to an opaque, black or reflection state. These metal depositions (plating) and dissolutions (depleting) can be reversibly achieved by applying reductive and oxidative voltages to the metal deposit, respectively. Unlike conventional ECDs, it has no fixed electrochromic layer and is, therefore, simpler in structure. Figure 2c shows the common structure and operating principal diagram of an RME. The most widely studied metals for RME are Ag, Bi and Cu [81,82,83]. Although Au [84], Zn [85], Pb [86] and other metals can be applied, their poor reversibility or the need for hazardous electrolytes lead to rarely reported. This kind of device is ideal for achieving light and teat modulation because a thin metal film with a thickness of a few tens of nanometers would be highly reflective in the visible and infrared regions [87,88]. In recent years, it has been widely applied in displays, smart windows, and thermal camouflage. As smart windows always require a large transparent area, a lower switching speed but a much larger working area are being investigated. Since metal materials form limited growth sites during growth, thereby forming a coarse and non–reflective metal layer that is highly absorptive because of the localized surface plasmon resonance (LSPR) effect and appears to be different colors [89]. This device still has room for progress in electrolyte design due to the existence of irreplaceable electrolytes. Moreover, the achievement of separate control of the radiation originating from different spectrum regions presents a difficult but interesting issue in the future.

Since the device structures and application directions differ between the different types, we focus our attention on their performance differently.

## 3. Performance Parameters of ECDs

### 3.1. Switching Time and Response Rate

Switching time is defined as the time needed for an ECD to switch between two different states (bleached and colored). It is an important parameter to evaluate the response–ability. The value of it is usually equal to the time it takes for the optical contrast to change from initial to 90%/95% during the coloring or fading process of the ECD. There are many factors affecting this performance, which are related to the electronic conductivity of the electrodes, device structure, functional layer material and excitation voltage, in addition to the ionic conductivity of the electrolyte and the ionic diffusion process of the device. The switching time requirements for the devices vary in different application directions. For example, as a display device, the switching time needs to reach the millisecond level, but as a smart window for an automotive canopy, the switching time can reach the minute level.

However, as far as we know, there is a significant difference in the variation of optical properties between devices, so it is not fair for us to discuss the responsiveness between different responses in terms of switching time only. Therefore, the response rate can be used in another way. The response rate (*v*) is the change in optical absorption (Δ*A*, or transmittance Δ*T*) of the device per unit time, in which *T* is the switching time, as shown in Equation (1).

(1) $$v=\frac{\Delta A}{T}\;\mathrm{or}\;v=\frac{\Delta T}{T}$$

Many factors affect the response time of the device, including the surface morphology of the device, the applied voltage, the cation radius in the electrolyte, the activation factor content, etc.

### 3.2. Optical Modulation and Contrast Ratio

Both optical modulation and contrast ratio are the main parameters for an ECD to evaluate its potential applications. Furthermore, they both refer to the difference between the colored and bleached states in terms of transmittance or absorbance at a specific wavelength. The difference is that optical modulation (Δ*T* or Δ*A*) is the change of them, and contrast ratio (CR) is a ratio of Equations (2) and (3):

(2) $$\Delta T=T_{coloured}-T_{bleached}\;\mathrm{or}\;\Delta A=A_{coloured}-A_{bleached}$$

(3) $$CR=\frac{T_{coloured}}{T_{bleached}}\;\mathrm{or}\;CR=\frac{A_{coloured}}{A_{bleached}}$$

### 3.3. Colouration Efficiency

Coloration efficiency (CE) is a practical parameter to measure the power requirements for the colored process. It is defined as the optical density or absorbance (Δ*OD*/Δ*A*) change of a specified wavelength induced by the injected charge per unit area (*Q_d_*). It accounts for the charge requirement of electrochromic switching. A higher CE means better electronic utilization efficiency. It is usually measured at the wavelength of maximum absorbance, *λ_max_*. Moreover, the value of it can be converted into the change of transmittance according to the Beer–Lambert law:

(4) $$CE\left(\eta \right)=\frac{\Delta OD}{Q_{d}}\;\mathrm{or}\;CE\left(\eta \right)=\frac{\Delta A}{Q_{d}}=\frac{\log\left(\frac{T_{bleached}}{T_{coloured}}\right)}{Q_{d}}$$

where *Q_d_* is expressed as charge density (C/cm^2^), *T_bleached_* and *T_coloured_* are the transmittances of the bleached state and the colored state.

Based on the definition of CE, the value of it at various optical change degrees can be directly calculated and usually obtained at the 90%/95% of the total optical change. Because the remaining percent color change (5%/10%) that takes place much more slowly should not contribute to a significant visually perceptible difference [90]. If the CE calculation includes the complete (100% *T_bleached_*) switch, the performance will be underestimated and detached from the actual observation [91]. In recent years, researchers have been working on the problem of improving the coloring efficiency of devices, and recently it was found that the introduction of a small number of rare earth elements in thin films can effectively improve the coloring efficiency of devices [92].

### 3.4. Cycling Stability

The cycling stability represents the service life of an ECD, which is measured by the change of density or the optical change after coloring–bleaching cycles. It is the most important property that needs attention in the application of ECDs. In addition to the cycling stability of the functional layer material, the device structure, environmental conditions and applied voltage also have an important impact on the stability of the device. In practical applications, the cycling stability of the device is required to maintain excellent electrochromic performance after 10^4^–10^6^ cycles, which is the main obstacle to the further development of most devices.

As we know, organic polymers are not comparable to inorganic electrochromic materials in long–term stability, though. However, they have more outstanding performance in terms of response speed, coloring efficiency, and color change diversity [93]. Organic conjugated polymers are one of the important electrochromic materials. Therefore, how to improve the long–term stability of organic conjugated polymer electrochromic materials has received more and more attention from researchers in recent years. Bo H. et al., Lawrence Berkeley National Laboratory of the United States, reported that an electron acceptor material bay–annulated indigo (BAI) could achieve high cycling stability of 7500 cycles after device preparation [94]. Wei TN. et al., Agency for Science, Technology and Research of Singapore, introduced fluorine atoms into the conjugated polymer backbone, which greatly enhanced the environmental stability through the fluorination reaction of electron acceptors and cycled between +1.6 V and −1.6 V for 10,000 cycles with no significant degradation [95]. This work by improving the environmental stability and cycling stability of organic materials is very meaningful and can promote the device to broaden the use of the device based on the short response time, high coloring efficiency, low turn–on voltage, and rich color of organic electrochromic materials.

An effectively rolled–out ECD should be able to maintain excellent performance over several years. One of the environmental conditions that most directly affects it is temperature. It should remain stable in both supercooled and superheated environments. In addition, to improve the lifetime of the device, sealed devices can be used to avoid the adverse effects of air and water in the external environment, as well as the temperaturedependent voltage application and reasonable electrochromic materials we mentioned above.

In recent years, many efforts have been made by researchers to develop highly durable, thermally stable, and efficient electrochromic devices. In 2019, Masayoshi H. and Sanjoy M. et al., National Institute for Materials Science of Japan, a solid–state ECD with high thermal durability were reported. The cathode coloring layer is Fe(II) fund belonging to polymer (polyFe), and the anode is Prussian blue as well as a poly (methyl methacrylate)–based solid–state electrolyte layer. The device has good transmittance regulation and a short response time, and more importantly, it can maintain 50% of the original performance under the high–temperature condition of 100 °C [96].

### 3.5. Memory Effect

In most cases, a constant supply of electric current is required to sustain a certain color associated with an electro–oxidized or–reduced state. There are, however, some materials that require almost zero–current consumption to maintain a certain color state, which is known as the “memory effect”; it is also known as bistability. Since it does not require a continuous power supply, some devices have been applied in the direction of electronic paper, etc. To achieve a more optimal memory effect and stabilize the redox state of the electroactive material inside the device, a complex structural design of the device is required. One of the most useful approaches is to rationalize the design of the electrolyte to reduce the free diffusion and exchange of electrons inside the device and to avoid the self–cancellation phenomenon. For example, replacing the liquid electrolyte of the device with a solid or semi–solid one.

In summary, the properties and characteristics of the recently reported electrochromic materials are shown in Table 2.

## 4. Effect of Temperature on Device Surface Morphology and Crystal Structure

### 4.1. Morphology of Electrochromic Materials

The main reasons for the influence of morphology on the performance of ECD are (1) when the roughness of the film is larger, the effective area of contact between the ions in the electrolyte and the film increases, while the distance of ion migration is reduced. It enhances the coloration efficiency of the device, speeds up the response rate and improves the optical contrast. (2) For RME devices, when the deposited film roughness is small, the metal film grows more uniformly and densely, which reduces the light absorption and transmission caused by defects and enhances the reflectivity of the film. This allows for better use of reflective devices. Many factors affect the surface morphology of thin films, such as the preparation method, annealing temperature, and addition of additives. Researchers have been well–established in this area in recent years. The influence of the preparation process is well summarized in other reviews [120,121,122], and we will not repeat it here. Better–performing films are obtained by adding organic additives to induce the directional growth of electrochromic materials [123,124]. Alternatively, to obtain a flatter film after deposition and to improve its reflectivity [125,126]. In addition, the film’s flatness can be improved by increasing the growth time to enhance the reflectivity [127].

**Figure 4:**
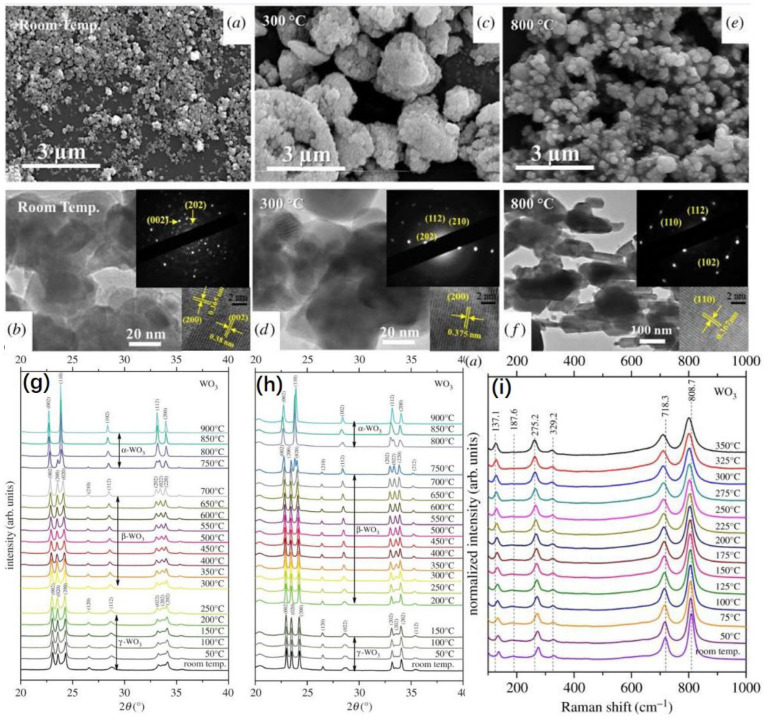
(**a**–**f**) show SEM and HRTEM images of the WO3 nanoparticles after treatment at various temperatures. The in situ XRD during (**g**) heating and (**h**) cooling and (**i**) in situ Raman spectroscopy of WO3 in different temperatures. Reprinted with permission from Ref. [130]. Copyright 2018, Royal Society Open Science. More details on “Copyright and Licensing” are available via the following link: https://www.mdpi.com/ethics#10.

The effect of temperature on the surface morphology of the film can directly lead to a change in the electrochromic properties of the device. On the one hand, the crystal structure of the material changes as the annealing temperature increases, causing the lattice to expand, resulting in an increase in grain size and a change in film roughness. However, the relationship between this change in roughness and temperature is not monotonic, so we need to determine its optimal growth temperature experimentally [128,129]. Figure 4a–f shows SEM and HRTEM images of the WO_3_ nanoparticles after treatment at various temperatures. The change in temperature can affect the morphology and crystal structure of thin films. On the other hand, for RME, the temperature has a significant effect on the shape of the electrochemically deposited film.

**Figure 5:**
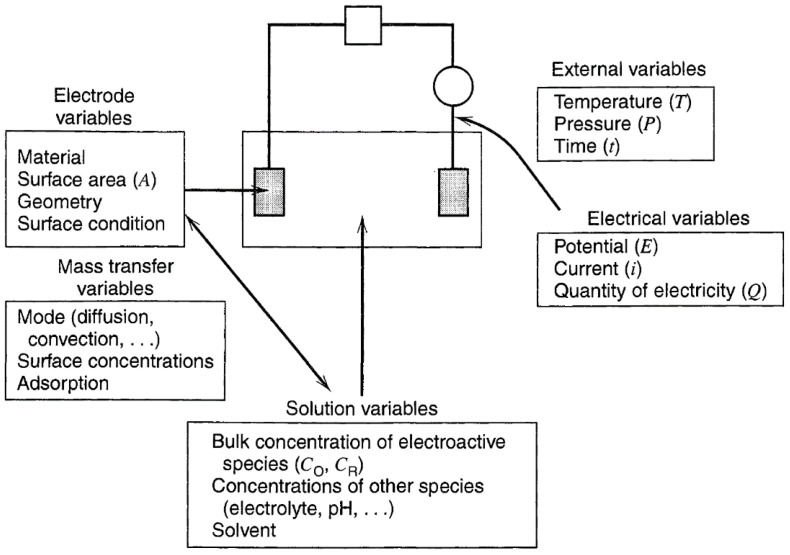
Variables affecting electrochemical phase formation. Reprinted with permission from Ref. [140]. Copyright 2001, Wiley. More details on “Copyright and Licensing” are available via the following link: https://www.mdpi.com/ethics#10.

To discuss the effect of temperature on electrodeposited films, it is first necessary to have an understanding of the electrochemical variables that may contribute to this factor. The important parameters in the electrolytic cell are shown in Figure 5. As an example, the work of Christopher et al. focuses on the ability of a 25 cm^2^ device to operate properly in −40 °C ambient conditions due to electrolyte conditioning [131]. This provides us with an idea to study the improvement of the device operating stability. However, this work does not account for the effect of temperature on the morphology of the deposited films. In other literature, we found different results of decreasing deposition temperature on thin films with porous [132] and dense [133], grain refinement [134,135] and coarsening [136,137], so the thermodynamic and kinetic effects of temperature driving on phase formation are of great interest to study. Many factors affect the thermodynamics and kinetics of electrochemical crystallization, among which the presence of an energy potential barrier makes nucleation a one–box probability event, which has the rate shown in Equation (5):

(5) $$j_{0}=Z_{0}W\lambda^{-1}exp\left[\frac{-\Delta G_{crit}}{kT}\right]$$

where Z_0_/cm^−1^ = the number density of active sites on the substrate, W/s^−1^ = the frequency of attachment of single atoms to the nucleus, *λ*^−1^ = a nondimensional quantity accounting for the difference between the quasi–equilibrium and the stationary number of nuclei, Δ*G*(*n_c_*) = the maximum energy barrier at a critical cluster size *n_c_*, the *k* and *T* is their usual meanings. The potential barrier to be overcome for atom formation is a temperature–dependent function, so changing the temperature affects the kinetic process of the reaction. This is because lowering the temperature leads to an increase in supersaturation, which increases ionic activity and generates critical nucleation conditions. When the cluster size exceeds the critical size, the growth kinetics can be expressed as Equation (6):

(6) $$j=\nu_{SL}S_{crit}C_{n}=\nu exp\left[\frac{-\Delta H_{d}}{kT}\right]S_{crit}C_{l}exp\left[\frac{-\Delta G}{kT}\right]$$

where $\nu_{SL}$ = jump frequency of atoms forming the liquid to the critical cluster (It can be estimated from lattice vibration frequency ν and activation energy barrier for interfacial diffusion Δ*H_d_*), *S_cr_* = the number of atoms surrounding a cluster, *C_n_* = the number of critical clusters. The increase in the number of critical clusters when the temperature decreases are due to the increase in the diffusion potential barrier, which decreases the adhesion rate of atoms to clusters and leads to a decrease in the crystal size.

### 4.2. Structural of Electrochromic Materials

In this section, we focus on the temperature response of the electrochromic and ion storage layers to temperature. They are mainly found in inorganic and organic electrochromic systems due to different device structures. This mainly consists of two parts; one is the effect of annealing temperature on the film structure, and the other is the effect of operating temperature on the film structure.

The relationship between annealing temperature and the structural, morphological, optical, and electrical properties of the film is quite tight. Post–annealing at different temperatures will affect the transportation of ions and electrons as it is highly dependent on the change in crystallinity of the films. Therefore, in ECDs application, the post–annealing process significantly affects the EC performance of different films.

On the other hand, operating temperature is also an important factor for the structure of electrochromic materials. This is very relevant to the temperature–dependent structural stability of the materials. Taking WO_3_ as an example, the ε–WO_3_ exhibited a monoclinic phase at less than −43 °C; the δ–WO_3_ showed a low temperature (−43 to 17 °C) triclinic phase with the $P\bar{1}$ space group, the γ–WO_3_ exhibited a room temperature stable monoclinic phase with P2_1_/n space group, and the β–WO_3_ showed orthorhombic phase at 300–700 °C, the α–WO_3_ showed tetragonal phase at greater than 740 °C [130,138,139]. In Figure 4g–i is the in situ XRD and in situ Raman spectroscopy of WO_3_ in different temperatures [130]. As the temperature changes, the crystalline phase of WO_3_ transforms with it. Even when no crystalline phase change occurs in a smaller temperature range, the crystal structure is dominated by subtle differences due to vibrations related to the stretching (i.e., stretching and bending) of the bonds themselves. The increase in operating temperature thus leads to a change in the width of the tunnel formed between the octahedra, making it easier for the ion embedding/detachment reactions to take place in the electrolyte.

## 5. Effect of Temperature on the Energy Required for Electrochemical Reactions

Electrochromism is an electrochemical reaction, and the vast majority of electrochemical experiments are performed at room temperature. The extended temperature range has a large impact on the application of substance transport phenomena and reaction kinetics.

The main effect of increasing the temperature on the transport of substances is to increase the mass transport to the surface of a large number of electrodes by diffusion and convection. This part of the work focuses on the temperature dependence of the activation energy of coupled homogeneous chemical reactions such as diffusion coefficient, diffusion activation energy, and in–bond cleavage. The two most important equations that can relate diffusion data to temperature changes are the Arrhenius plot (Equation (7)) and the Stokes–Einstein equations (Equation (8)):

(7) $$\ln D=\ln D_{0}-\frac{E_{A}}{RT}$$

(8) $$D=\frac{k_{B}T}{6\pi \eta r}$$

where *D* = diffusion coefficient, *E_A_* = activation energy, *R* = molar gas constant, *T* = temperature, *k_B_* = Boltzmann constant, η = solvent viscosity, *r* = hydrodynamic radius. Moreover, the activation energy of diffusion for a given substance can be found by a plot of ln *D* against 1/*T*, or a plot of *D* against mnm*T*/η can be used to determine the effective radius of a species in solution.

Theoretical expressions for diffusion coefficients have been developed as the most commonly used electrochemical methods and can be easily obtained from steady–state or transient current measurements. The most common method is the microcurrent method, where the limit current is expressed as Equation (9):

(9) $$I_{Lim}=4nFDc_{\infty }r$$

where *n* = number of electrons transferred, *F* = Faraday constant/C mol^−1^, *D* = diffusion coefficient/cm^2^ s^−1^, *c*_∞_ = bulk concentration of the solution/mol cm^−3^, *r* = radius of the electrode/cm. When the radius and temperature of the microelectrode are known, the limiting currents can be measured for various concentrations of a one–to–one substance in solution. The diffusion coefficient at that temperature can be obtained by plotting of *I_Lim_* against *c*_∞_ under isothermal conditions. The experiment is then repeated at different temperatures, and the obtained diffusion coefficients can be used to construct Arrhenius plots. In addition to this, the Arrhenius equation can be calculated using the electrical conductivity of a solid at different temperatures.

(10) $$\sigma =\sigma_{0}exp\frac{-E_{a}}{kT}$$

where σ is the diffusion coefficient (Ω/m); *E_a_* is the activation energy; *T* is the absolute temperature in the unit of Kelvin (K) at which the reaction takes place; *k* is the Boltzmann constant (8.617 × 10^−5^ eV/K). For convenience to interpret graphically, the equation can be written in a nonexponential:

(11) $$\ln\sigma =\ln\sigma_{0}-\frac{E_{a}}{kT}$$

**Figure 6:**
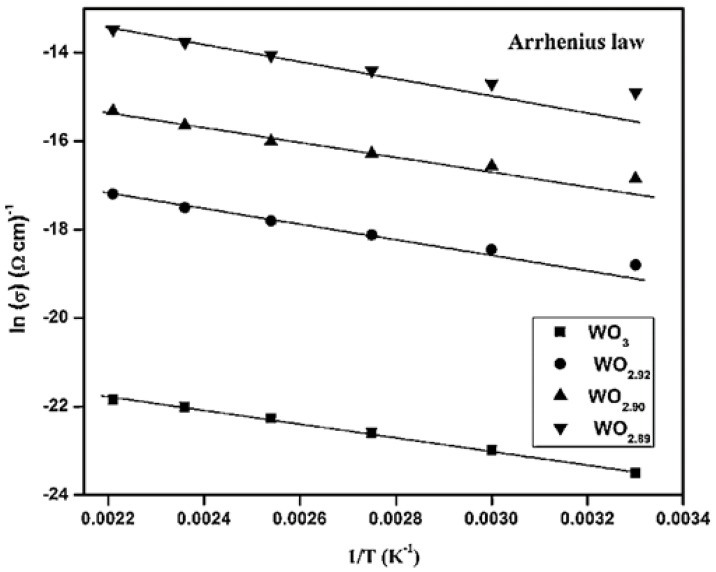
Arrhenius plot of ln(σ) versus 1/T for WO_3_, WO_2.92_, WO_2.90_, and WO_2.89_ thin films. Reprinted with permission from Ref. [142]. Copyright 2016, Elsevier. More details on “Copyright and Licensing” are available via the following link: https://www.mdpi.com/ethics#10.

The negative activation energy divided by the Boltzmann constant is equal to the slope of the Arrhenius plot. The activation energy is the initial energy that the electric charges need to move inside the material and is equal to the potential barrier heights between the crystallites [141]. It can be seen from Figure 6 that the activation energy is not single in the whole temperature interval, and the activation energy is different in different temperature regions [142].

The coupled homogeneous chemical reactions are temperature dependent, and therefore the chemical reactions are required for the activation energy to proceed. Thus, chemical reactions can drive the reaction process by increasing the temperature when the progress is slow at room temperature. In addition to Arrhenius plots, we can also simulate the reaction system by the activation energy of a chemical reaction at variable temperatures can be obtained by calculating the reaction at isothermal or unequal temperatures, respectively, and giving the constant value at room temperature in the case of Digisim [143] or FIDAP (A Fluid Dynamics Analysis Program) [144], which are suitable for this analysis.

Elevated temperatures and transport of elevated substances by diffusion or convection are often evident in the electrical signal. However, due to factors such as background noise, there are also higher requirements for the sensitivity of the monitoring equipment.

The change in activation energy required for the electrochemical reaction has an impact on the actual turn–on voltage required for the device to operate at different temperatures. In real applications, ECDs are used in the temperature range of −40 °C to 80 °C. Furthermore, at this time, still at room temperature, a switching voltage may be applied to the device overvoltage (or undervoltage), which will greatly reduce the life of the device and optical performance. This is because the magnitude of the voltage that turns the device on is temperature–dependent, requiring a lower voltage at higher temperatures [10]. The temperature has an exponential relationship with the diffusion coefficient of ions, and when the temperature increases, the ion diffusion will increase significantly and decrease the switching voltage [145].

To address this adverse effect, we should focus mainly on exploring the voltage–temperature functions of different ECDs and using the model to optimize the matching voltage output to extend the device lifetime as much as possible in the future. On the other hand, it can be started to improve the ionic conductivity of the electrolyte and reduce the interfacial barrier of ion insertion–deconstruction, to obtain a smaller switching voltage and reduce the effect of temperature on it. We can currently follow the ion embedding and disembedding process through cyclic voltammetry, chronoamperometry, and chronopotentiometry. The electrochemical impedance method is used to determine the ion mobility of the electrolyte, the charge transfer process, and the resistive barrier present in the system. Adding a test dimension to the above in future studies is temperature–dependent electrochemical testing [2,146,147].

## 6. ECD Regulation of Environmental Temperature

**Figure 7:**
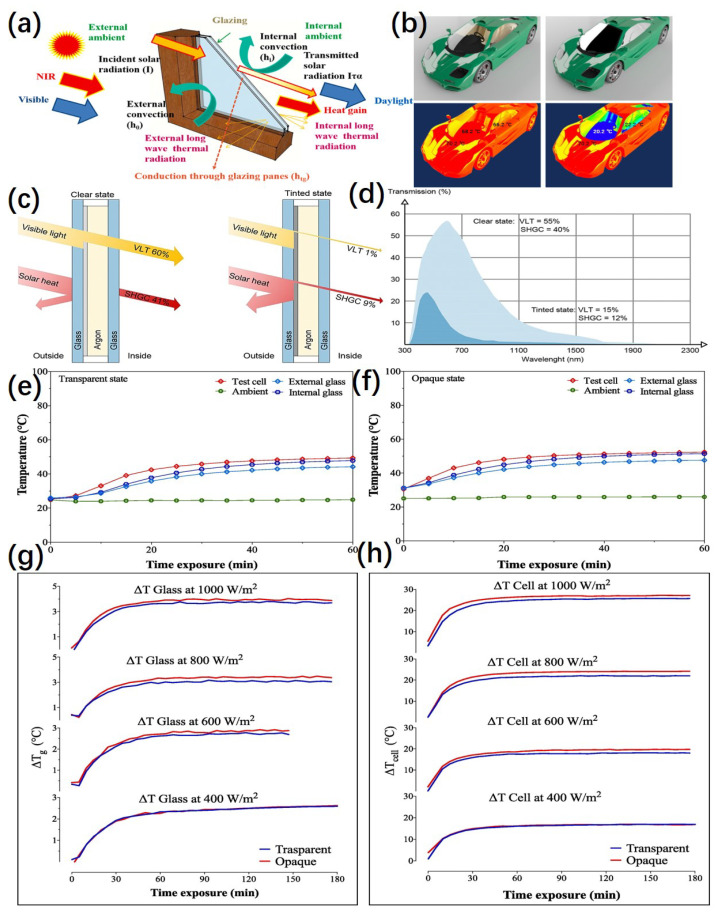
(**a**) schematic of a conventional window showing heat loss, heat gain and daylighting penetration. Reprinted with permission from Ref. [69]. Copyright 2021, Elsevier. More details on “Copyright and Licensing” are available via the following link: https://www.mdpi.com/ethics#10. (**b**) Simulation of infrared reflection effect of vehicle electrochromic glass based on RME device, (**c**) electrochromic glazing control states and (**d**) spectral transmission of electrochromic glass in different tint states. Reprinted with permission from Ref. [151]. Copyright 2018, Elsevier. More details on “Copyright and Licensing” are available via the following link: https://www.mdpi.com/ethics#10.The measured temperature of external and internal glazing surface, test cell and ambient environment in the (**e**) translucent state and (**f**) transparent state under 1000 W/m^2^ of PDLC. (**g**) the time variation of the temperature difference (ΔTg) between the internal and external across all various s radiation intensities. (**h**) the time variation of the temperature difference (ΔTcell) between the internal cell temperature and external ambient across all various radiation intensities. Reprinted with permission from Ref. [66]. Copyright 2020, Elsevier. More details on “Copyright and Licensing” are available via the following link: https://www.mdpi.com/ethics#10.

Smart windows can control the increase or decrease of heat (cooling and insulation) as needed, thus reducing energy consumption for temperature regulation, and are therefore considered a key component of energy–efficient, green building technology [148]. Figure 7a shows the schematic of a conventional window showing heat loss, heat gain, and daylighting penetration. It is mainly the transmission and absorption of infrared wavelengths by the ECD that causes changes in the room temperature to occur. Transmission is the main component, but absorbed solar energy can also be transmitted inward by conduction, convection and radiation [149]. Therefore, we can quantify the proportion of solar radiation entering the building to the total incident radiation by the solar heat gain coefficient (SHGC). The SHGC is calculated by measuring the total heat flow using a calorimeter chamber [150] or simulations [126], and values of 0.6 and 0.2 are usually recommended for winter and summer as general guidelines. Dynamic smart windows cause better regulation of SHGC and visible light than traditional static windows that only control heat loss from inside to outside. Solar heat gain alteration using ECD that can bring about this change would be highly desired. ECD control SHGC and daylight mainly include transition metal oxide systems, liquid crystal–based electrochromic systems, and reversible metal electrodeposition–based electrochromic systems. For example, its application in vehicle–mounted intelligent Windows. Figure 7b shows the simulation of the infrared reflection effect of vehicle electrochromic glass based on the RME device.

ECDs with WO_3_ and VO_2_ as functional layers have been of great interest in the regulation of IR band transmittance. In 2018, Casini [151] prepared a complete review of the active dynamic windows available on the market, which contains a complete description of the major active color development technologies, including commercial ECD. Figure 7c,d shows the electrochromic glazing control states and spectral transmission of electrochromic glass in different tint states. In this paper, the authors give examples of different techniques and the five–layer architecture chosen to analyze the transition metal oxide electrochromic system with WO_3_ as the anode and VO_2_ as the cathode. The SHGC of this device can be adjusted from 0.41 in the bleached state to 0.09 in the colored state, with T_vis_ passing from 60% to 1%, requiring very minimal power, about 2.5 W/m^2^. This kind of commercial product offers 10 years warranty and 30–year service life.

We should first analyze PDLC thermal performance before the SHGC of it. In 2020, an Evaluation of the thermal properties of polymer–dispersed liquid crystal (PDLC) glass was reported by Abdulmohsin et al. [66]. In this paper, PDLC glass was exposed to a constant indoor solar simulator for 180 min at different radiation intensities (1000, 800, 600, 400 W/m^2^) in transparent and translucent states. The PDLC system was observed to exhibit similar behaviors at different radiation intensities.

Figure 7e,f shows that during the transparent state under the radiation of 1000 W/m^2^, the test cell temperature increased from 27.3 °C to 51.22 °C, but the ambient form 24.00 °C to 25.54 °C. It was determined that PDLC indicates higher energy transmission and greater heat flow. Figure 7g shows the time variation of the temperature difference (ΔT_g_) between the internal and external across all various s radiation intensities. It was also found that the translucent state has higher temperature differences than the transparent state. This indicates that the PDLC system exhibits higher absorption levels of radiation rather than reflection. Figure 7h shows the time variation of the temperature difference (ΔT_cell_) between the internal cell temperature and external ambient across all various radiation intensities. It was determined that the overall heat flow to the test cell through the PDLC glazing in the translucent state is higher compared to the transparent state. The SHGC of the PDLC can be adjusted from 0.68 in the transparent state to 0.63 in the translucent state. It has similar SHGC values in both states, and the modulation ability for SHGC is not obvious, which is due to the lower ability of PDLC for reflection and transmission of the sun in both states. However, it is also due to this property that PDLC glass is suitable for cold climates.

RME has a higher transmittance modulation capability because they do not require a thicker oxide layer and therefore reduces the absorption of visible light, similar to the modulation of SHGC [13,152]. Michael and colleagues in the USA report on the addition of polymer inhibitors to the electrolyte that improves the optical and solar performance as well as the durability of an RME [126]. The addition of polyvinyl alcohol (PVA), an inhibitor of the plating process, controls the growth morphology of the metal film. The smooth and dense metal film effectively enhances reflectivity, reduces scattering, and lowers absorption. Thus, enabling the device to be optically modulated over a wide spectral range. In the visible band, the difference in transmittance between the transparent and reflected states of the device is 76%, and the lowest transmittance can be as low as 0.001%. The researchers completely calculated the SHGC of RME. The SHGC is from 0.60 to 0.04 when the device is switched between transparent and reflective states. Such large modulation is a result of the highly visible transmittance of the device in the transparent state and the reflective nature of the RME technology. Being able to modulate the SHGC over a wide range is important to control the heating and cooling loads associated with the device [153].

For the existing technologies for the regulation of temperature by smart windows, RME technology causes the largest SHGC and is the most promising. Nevertheless, at the same time, the ECD of metal oxide materials, although the SHGC is reduced due to the absorbed thermal radiation, is currently of great interest due to its good thermal stability, cyclic stability and ease of mass production. PDLC is suitable for colder regions where the main focus is on raising the temperature due to its larger transmission and absorption making its higher SHGC.

## 7. Conclusions and Prospects

ECDs are powerful light modulation and thermal management devices due to their outstanding spectral control ability, switching speed, high contrast, and low operating voltage in the visible and near–infrared fields. Although the devices have been optimized in terms of materials and performance after decades of research, there are still several issues that need to be addressed in terms of higher requirements for device performance in specific applications, as follows.

1. Short cycle life. Ions in the color–changing material embedding off incomplete, resulting in the device’s color contrast being reduced when the period is reduced to 90% or 95% or less to determine the device failure. In addition, there are also problems such as irreversible damage caused by the ion radius being too large and the film peeling off due to the multiple embedding and detachment of the multicolor change layer film, making the device fail. The rational design of the device structure and the design between the electrolyte and the color–changing film layer are also important. Electrode failure, electrolyte decomposition and side reactions during the color change process are also the reasons for the short lifetime of ECDs. For applications such as displays and electronic paper, the device life should be more than 10^9^, and for smart windows, heads–up display glass and other devices with a service life of more than 10 years, the cycle life should be more than 10^4^ times, while most of the reported device cycle life is still in the order of 10^3^. Therefore, it is important to improve the cycle life of the device for a wide range of applications.
2. Difficulty in preparing large area devices. The main problem with this part is that the high transparency and high conductivity of the electrodes cannot be obtained at the same time. When the conductivity of the substrate is low, there is a large potential difference between the center and the edge of the device. To ensure the color change effect in the middle position, a large working voltage must be used, which leads to unstable electrodes, uneven electrolyte decomposition and poor reversibility of the color change effect, as we mentioned earlier, affecting the device lifetime.
3. Unanticipated side effects. To improve the device color contrast and response time and other performance, we hope that the color–changing material layer has a larger active specific surface area and higher electrochemical reaction activity, but also, because of this often occurs some side reactions. For some inorganic EC materials, the color change process is often accompanied by some unwanted catalytic reactions. It is well known that the response rate of ECD is proportional to the contact area between the electrode, electrolyte and electroactive material, but unfortunately, it is also usually proportional to poor interfacial resistance and side reactions. Therefore, new electrochromic materials are developed, and other improved solutions are used to obtain very good durability and fast switching speed. This approach has been explored by many researchers, but the results are still unsatisfactory.
4. High cost of material preparation. Lower preparation cost and a simpler preparation process for high–quality ECDs is the key to their eventual commercialization. Although the price of electrochromic smart windows is decreasing year by year with the development of preparation technology, this technology is a luxury rather than a critical feature compared to standardized transparent glass. Therefore, how to enhance the additional functional attributes of ECDs while reducing the device preparation cost is also an option to be considered, such as some head–up displays, self–functional ECDs and other related research.

To solve the above problem, there are several possible ways to achieve this goal, as shown in the following. First, it is essential to have an in–depth understanding of the reactions and side reactions in the electrochromic process. We need to know the exact molecular structure of the by–products and to grasp what causes the by–reactions. Secondly, Optimizing the transparent electrode design and preparation process to enhance the transmission rate of the electrode while reducing the voltage reduction of the electrode is one of the solutions for large–area and large–scale production. The compatibility of the processing and preparation of each film layer of the device is also considered. However, we believe that with the rapid development of material science, better development in the preparation process and component compatibility of devices will be achieved in the future. Finally, to carry out studies related to the effect of the operating environment on device performance, such as temperature, light, water/oxygen, atmospheric pressure, etc. In this way, we can avoid the occurrence of side reactions by changing the working environment and reaction conditions of ECDs. Although this part of the work has been partially studied, it is still in the primary research stage, and no systematic research results are available. Second, reducing the manufacturing cost of materials, films and devices is the key to the eventual commercialization of ECDs. There are many methods for the preparation of electrochromic materials, such as the sol–gel method, hydrothermal method, electrostatic spinning, etc., but how to guarantee high–quality preparation while reducing the cost of mass production is still a problem for all researchers. In addition to the preparation technology of thin film materials, there is a need to optimize the corresponding film–forming technology to prepare high–performance ECDs. The birth of some new processes in recent years gives us hope, such as automatic blade coating, roll–to–roll layer and inkjet printing technologies, which are more suitable for application in a large area and low–cost manufacturing compared with traditional magnetron sputtering, vacuum evaporation and rotary coating technologies.

Fortunately, many brilliant researchers are doing their best to solve these problems that plague us. It is conceivable that we will have better solutions and better–performing ECDs shortly, and the problems we are discussing may also stimulate research and development in other areas of color–changing glass and achieve better results in other optoelectronic fields.

## Figures and Tables

**Table 1:** Colors and type of coloring of transition metal oxide electrochromes.

Materials	Colour in Oxidized from	Colour in Reduced from	Type of Colouring
WO_3_ [19,20,21]	Pale yellow	Dark blue	Cathodic
TiO_2_ [22,23,24]	Colourless	Blue	Cathodic
Nb_2_O_5_ [25,26]	Colourless	Brown–black	Cathodic
MoO_3_ [27,28]	Colourless	Dark blue	Cathodic
Ta_2_O_5_ [29,30]	Colourless	Black	Cathodic
ZnO [31,32,33]	Colourless	Blue	Cathodic
NiO [34,35]	Brown/black	Colourless	Anodic
IrO_2_ [36,37]	Blue–black	Colourless	Anodic
MnO_2_ [38]	Dark brown	Pale yellow	Anodic
Co_3_O_4_ [39,40,41]	Blue	Brown	Anodic
Cu_2_O [42,43]	Black	Metallic reddish–brown	Anodic
V_2_O_5_ [44,45]	Brownish–yellow	Pale blue	Cathodic & Anodic

**Table 2:** Summary of the recent progress of electrochromic materials for ECDs.

Electrochromic Material	Switching Time	Colouration Efficiency (cm^2^ C^−1^)	Optical Modulation	Cycling Stability (Cycles)	Memory Effect
80% hydroxypropyl acrylate—20% methyl methacrylate copolymer [97]	7.5 s	872	>70%	1000	50 h
Electrochromic conjugated polymers [98]	≤2 s	---	60.6%	12,000	---
PANI/Au nanorods [99]	0.9 s	---	56%	6750	---
thermally self–healable polyurethane [100]	0.8 s	324.9	93%	100	---
Nb_18_W_16_O_93_ [101]	4.7 s	46.57	53.1%	8000	---
Cu/WOx^–^Al^3+^/GR [102]	140 s	36.0	41%	2000	---
di–heptyl viologen with graphene quantum dots [103]	6.2 s	66	60%	---	
di–pentyl viologen with graphene quantum dots [103]	4.4 s	143.9	53.4%	3000	---
1,2,4,5–tetrakis (4–carboxyphenyl) benzene esters [104]	<3 s	261.0	64.5%	2350	---
thienoisoindigo–based electrochromic copolymers [105]	0.35 s	433.4	82%	---	---
Fe(II) based metallo–supramolecular polymer [77]	0.78 s	242.2	90%	>100	32 min
D–A type EDOT–based monomers consisting [106]	0.5 s	427	> 50%	---	---
Prussian White [107]	2.5 s	149.3	>70%	10,000	---
Tungsten Oxide/Graphene Quantum Dot [108]	4.1 s	78	78.72%	10,000	---
p–extended viologens consisting of quinoxaline–based bridges [79]	0.46 s	334	82%	---	---
Zn–Fe Prussian blue [109]	3.9 s	---	60%	10,000	40 min
TiO2@Graphene/Prussian blue Core–Shell [110]	1 s	129.1	56.1%	1000	8.3 h
quinacridone dye [111]	<1 s	498	40%	50,000	---
Prussian blue [112]	2.3 s	67.23	60%	10,000	---
ammonium metatungstate and iron (II) chloride solution [113]	<10 s	160.04	57%	100	---
NW/P_2_W_17_/Cu (phen)_2_ [114]	2.9 s	50.4	43.7%	---	---
MoS2/WO3 nanocomposite [115]	20 s	67	59%	100	---
Preyssler–type polyoxometalates and W_18_O_49_ [116]	2.62 s	149.78	50%	500	---
3–methylthiophene with 3,4–ethylenedioxythiophene [78]	6 s	685	84%	---	---
Hf–doped WO_3_ [117]	1.28 s	161.87	75%	1000	---
Ti–Doping V_2_O_5_ [118]	1.4 s	96.1	57%	1000	---
reversible metal electrodeposition of Bi–Cu	3.1 s	10.98	80%	2500	---
antimony–doped tin oxide [119]	0.4 s	27	90%	1000	---
